# Listeriosis Associated with Stone Fruit — United States, 2014

**Published:** 2015-03-20

**Authors:** Brendan R. Jackson, Monique Salter, Cheryl Tarr, Amanda Conrad, Emily Harvey, Lisa Steinbock, Amy Saupe, Alida Sorenson, Lee Katz, Steven Stroika, Kelly A. Jackson, Heather Carleton, Zuzana Kucerova, David Melka, Errol Strain, Mickey Parish, Rajal K. Mody

**Affiliations:** 1 Division of Foodborne, Waterborne, and Environmental Diseases, National Center for Emerging and Zoonotic Infectious Diseases, CDC; 2 Food and Drug Administration; 3 Atlanta Research and Education Foundation; 4 Massachusetts Department of Public Health; 5 City of Northampton Health Department, Massachusetts; 6 Minnesota Department of Health; 7 Minnesota Department of Agriculture

On July 19, 2014, a packing company in California (company A) voluntarily recalled certain lots of stone fruits, including whole peaches, nectarines, plums, and pluots, because of concern about contamination with *Listeria monocytogenes* based on internal company testing (1). On July 31, the recall was expanded to cover all fruit packed at their facility during June 1–July 17 (2). After the initial recall, clinicians, state and local health departments, CDC, and the Food and Drug Administration (FDA) received many inquiries about listeriosis from concerned consumers, many of whom had received automated telephone calls informing them that they had purchased recalled fruit. During July 19–31, the CDC *Listeria* website received >500,000 page views, more than seven times the views received during the previous 52 weeks. However, no molecular information from *L. monocytogenes* isolates was available to assess whether human illnesses might be linked to these products.

In early August 2014, a two-enzyme pulsed-field gel electrophoresis (PFGE) pattern shared by three *L. monocytogenes* isolates from stone fruit associated with the recall was uploaded to PulseNet, the national molecular subtyping network for foodborne disease surveillance. Four human isolates with isolation dates during the period May 8–July 8, 2014 (Illinois, Massachusetts, and South Carolina) and August 28 (Minnesota) were identified that had PFGE patterns indistinguishable from isolates from company A stone fruit. Samples of stone fruits from company A collected after the recall yielded an additional 31 *L. monocytogenes* isolates, 22 of which were indistinguishable from the initial isolates by PFGE; three other PFGE patterns were identified that did not match any isolates from clinical specimens collected during May 1–August 31. Whole-genome sequencing (WGS) analysis by whole-genome multilocus sequence typing showed that isolates from the Massachusetts and Minnesota patients were highly related (<10 allele differences and <10 high-quality single nucleotide polymorphism differences) to the isolates from recalled stone fruits, whereas the Illinois and South Carolina isolates were not (Figure).

A review of the standardized *Listeria* Initiative exposure questionnaire (3) for the Massachusetts patient showed that organic nectarine consumption was recorded, although the form does not specifically ask about stone fruit consumption. A subsequent interview using a questionnaire with questions about stone fruits indicated that the patient consumed nectarines and peaches purchased from stores that sold company A stone fruit. Traceback using receipts and shopper card data indicated the patient’s family purchased recalled fruit. An interview with a family member of the Minnesota patient revealed that the patient consumed peaches from a store that received company A stone fruit; however, dates from receipts indicated that the peaches were purchased after the recalled fruit was reported to have been removed from the shelves. After removal of recalled fruit, the store received company A peaches that were not part of the recall as well as peaches from another California supplier. The South Carolina patient reportedly did not eat stone fruit before becoming ill. Family of the Illinois patient could not be reached for interview.

Strong evidence linked the Massachusetts case to recalled stone fruit, including food exposure interviews, receipt and shopper card data, and WGS results showing very high genetic relatedness between the patient’s isolate and isolates from nectarines. Consumption data and WGS results suggest that stone fruit was also the likely source of *L. monocytogenes* infection in the Minnesota case; however, the later dates of illness onset and fruit purchase suggest that the patient consumed stone fruit that was not included in the recall.

This is the first reported link between human listeriosis and stone fruit. WGS results provided a basis for focusing resources for extended case interviews and follow-up. Specifically, among cases that matched the recalled stone fruit by PFGE, WGS allowed differentiation between sporadic cases and cases associated with stone fruit consumption.

Reported listeriosis is much more common in pregnant women than in the general population and can cause major fetal and perinatal complications. Because of this higher risk, and partially in response to public concern stemming from these recalls, the American College of Obstetricians and Gynecologists issued guidelines for management of pregnant women with possible *L. monocytogenes* exposure (4). Although exposure to this recalled product was likely widespread, disease was very rare. Therefore, this recall and associated illness does not provide sufficient evidence to recommend that persons at higher risk for listeriosis (e.g., pregnant women, persons aged ≥65 years, and immunocompromised persons) avoid fresh stone fruits. However, it does support the need to understand risks associated with contaminated, ready-to-eat fresh fruit so that prevention strategies can be strengthened.

## Figures and Tables

**FIGURE:**
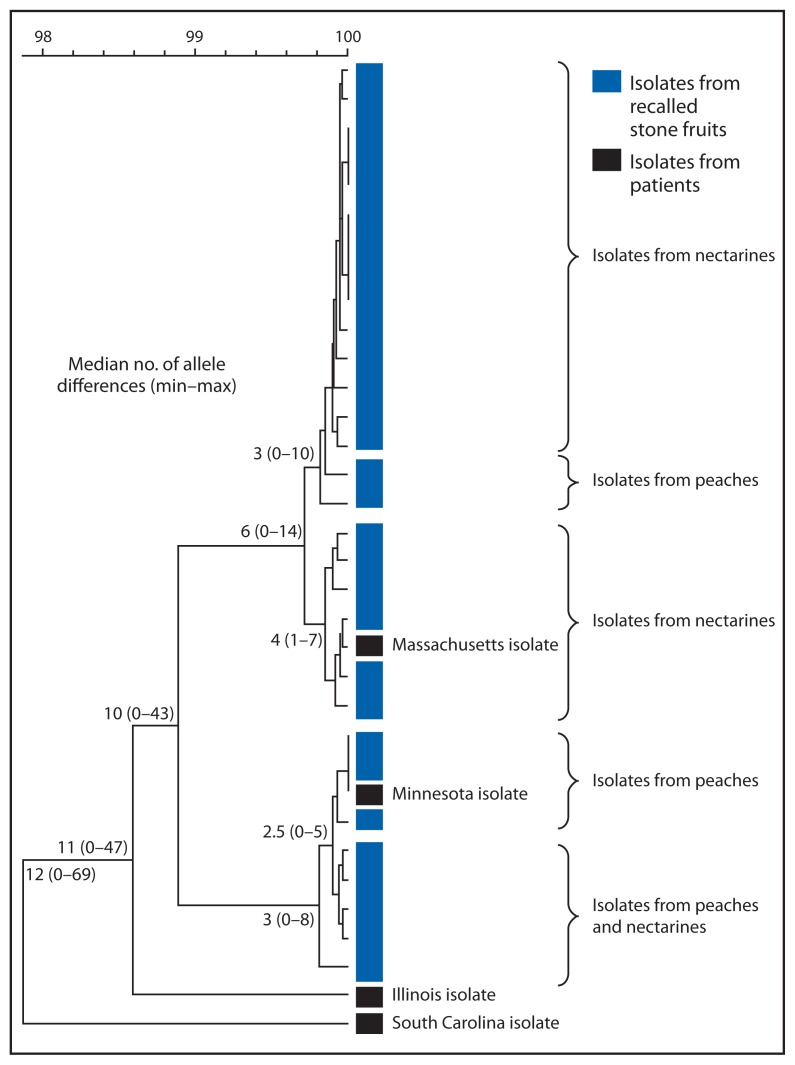
Phylogenetic tree by whole-genome multilocus sequence typing (wgMLST) of *Listeria monocytogenes* isolates from patients in four states and from recalled nectarines and peaches with indistinguishable pulsed–field gel electrophoresis patterns — United States, 2014* * By wgMLST, the Massachusetts patient isolate differed from six closely related nectarine isolates by ≤7 alleles, and the Minnesota patient isolate differed from three closely related peach isolates by ≤5 alleles out of >5,800 loci analyzed in BioNumerics 7.5 wgMLST analysis pipeline. The Illinois and South Carolina patient isolates differed from the most closely related stone fruit isolate by 47 and 69 alleles, respectively.
